# Motivation for people living with dementia to engage in research advisory boards

**DOI:** 10.1093/geroni/igab046.577

**Published:** 2021-12-17

**Authors:** Martina Roes, Dianne Gove, Ana Diaz

**Affiliations:** 1 German Center for Neurodegenerative Diseases (DZNE), Witten, Nordrhein-Westfalen, Germany; 2 Alzheimer Europe, Luxembourg, Diekirch, Luxembourg

## Abstract

The importance of Public Involvement (PI) is increasingly being recognized in the field of dementia research. In 2012, Alzheimer Europe set up the European Working Group of People with Dementia (EWGPWD) which provides advice and input for all activities of the organization including several large European-funded research projects. The German Center for Neurodegenerative Diseases (DZNE) created a research advisory patient board in 2020 with the intention of supporting the board in strategic research decisions. Both groups are composed of people with dementia and act independently. With the aim of finding out whether PI in research is mutually rewarding and beneficial, members of both groups were asked about their motivation to be involved in PI research activities and the value this had for them. This was collected either through narrative interviews or during meetings. People with dementia described several reasons for being involved in PI activities in dementia research.

